# A clinicopathological study of giant cell tumor of small bones

**DOI:** 10.3109/03009734.2011.596290

**Published:** 2011-10-29

**Authors:** Michiro Yanagisawa, Kyoji Okada, Takahiro Tajino, Tomoaki Torigoe, Akira Kawai, Jun Nishida

**Affiliations:** ^1^Department of Orthopedic Surgery, Hirosaki University Graduate School of Medicine, Hirosaki, Japan; ^2^School of Health Sciences, Akita University, Akita, Japan; ^3^Department of Orthopedic Surgery, Fukushima Medical University, Fukushima, Japan; ^4^Department of Orthopedic Surgery, Juntendo University, Tokyo, Japan; ^5^Department of Orthopedic Surgery, National Cancer Center, Tokyo, Japan; ^6^Department of Orthopedic Surgery, Iwate Medical University, Morioka, Japan

**Keywords:** clinicopathological study, giant cell tumor, p63 immunostaining, small bone

## Abstract

**Background and purpose:**

Giant cell tumor (GCT) of the small bones (small-bone GCT) is usually rare and considered somewhat different from conventional GCT. The purpose of this study was to investigate and report the clinicopathological features of 11 cases with small-bone GCT.

**Materials and methods:**

Patient information was obtained with the help of questionnaires. X-rays and paraffin blocks obtained from several institutions were clinically, radiographically, and histologically evaluated.

**Results:**

Small-bone GCT was observed in younger patients compared to conventional GCT; 5 of the 11 (45%) patients were below 20 years of age, whereas the corresponding figure for all GCT patients is 16% in Japan. Excessive cortical bone expansion is a special feature. There were two cases of recurrence and one case of lung metastasis; the primary lesion was in the hand for all three cases. In contrast, no primary lesion of the foot recurred or metastasized. Varying degrees of positive p63 immunostaining were observed in all examined cases (*n* = 9) of small-bone GCT but were negative in case of giant cell reparative granuloma (GCRG) and solid variant of aneurysmal bone cyst (ABC). One case that demonstrated high-intensity positive staining had two episodes of recurrence.

**Conclusion:**

Small-bone GCT tends to develop in younger patients than does conventional GCT. Primary GCTs of the hand may be biologically more aggressive than those of the feet. The p63 immunostaining may be useful not only for differential diagnosis but also for prognostication of small-bone GCT.

## Introduction

Giant cell tumor (GCT) is a relatively common type of benign bone tumor. Long tubular bones such as the femur, tibia, and humerus are commonly involved; however, the occurrence of GCT in the hands and feet is a rare phenomenon. It has been reported that GCT of the small bones (small-bone GCT) carries a higher risk of local recurrence and metastasis than conventional GCT. Although case reports of small-bone GCT have been reported frequently, few papers that describe clinicopathological features of small-bone GCT in a large series have been published. The purpose of this study was to investigate and report the clinicopathological features of small-bone GCTs and to identify previously unreported characteristics of the disease.

## Materials and methods

Clinical information on 11 cases of small-bone GCT was collected from several institutions using a questionnaire: X-rays, paraffin blocks, and non-stained glass slide specimens were also obtained from those institutions. The term ‘small bone’ was defined as a carpal bone or any bone distal to the carpal in the hand, and talus or any bone distal to the talus in the foot. The analyzed parameters included gender, age, affected bone(s), symptoms, primary surgical procedure, recurrence, and metastasis. Image evaluation was performed on the basis of the Campanacci stage classification (1). Hematoxylin-eosin (H-E) staining was performed for pathological evaluation. In addition, since p63 has been reported as a useful marker for the differential diagnosis of GCT (2,3), p63 immunostaining was performed to confirm the reliability of the initial diagnosis. The p63-positive cases were classified into three categories on the basis of the ratio of p63-positive nuclei among total mononuclear cells: slightly positive (≤5% positive nuclei); positive (5–30% positive nuclei); and highly positive (≥30% positive nuclei). The p63 immunostaining was also performed for specimens of other osseous tumors that were collected for the purpose of comparison. These included conventional GCT of the femur or tibia (*n* = 5), giant cell reparative granuloma (GCRG) of the metacarpal bone (*n* = 1), and solid variant of aneurysmal bone cyst (solid variant ABC) of the humerus (*n* = 1).

## Results

Information was obtained from five male and six female cases with a mean age of 24.7 years (range 9–60 years) at initial diagnosis. The bones affected were metacarpals (*n* = 5), metatarsals (*n* = 1), proximal phalanx of the hand (*n* = 1), proximal phalanx of the foot (*n* = 1), middle phalanx of the hand (*n* = 1), talus (*n* = 1), and cuboid (*n* = 1). Subjective symptoms at initial examination included pain only (*n* = 9), swelling only (*n* = 1), and swelling with pain (*n* = 1). With respect to radiographic staging, two, six, and three cases corresponded to stages 1, 2, and 3 of the Campanacci classification (1), respectively.

**Figure 1.**
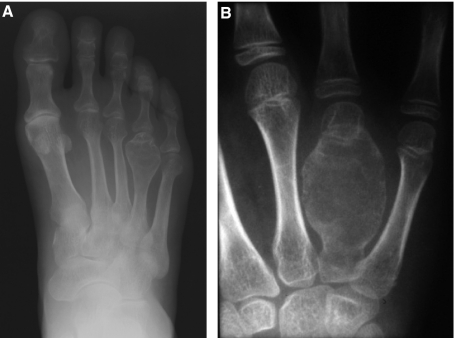
Radiograph of GCT involving the distal fourth metatarsal bone (A) and diaphysis of the fourth metacarpal bone (B). Both lesions are purely lytic with partially sclerotic rim and markedly expanded cortical bone, but with no cortical destruction. The centers of the lesions appear to be centrally located within the bones.

**Figure 2.**
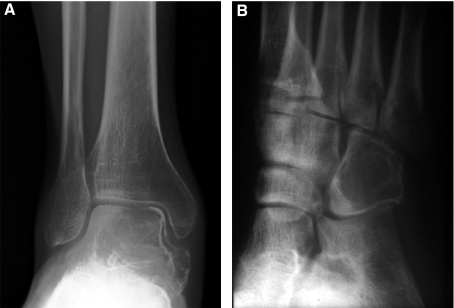
Radiograph of GCT involving the talus (A) and cuboid (B). As in Figure 1, both lesions are purely lytic with partially sclerotic rim, although the expansion of bone is not prominent. The lesions appear eccentric.

The radiographic appearances were confirmed in four short tubular bones and two tarsal bones. The distribution of the intraosseous lesions of the short tubular bone was as follows: two lesions were present between the epiphysis and metaphysis, one between the epiphysis and diaphysis (Figure 1A), and one in the diaphysis (Figure 1B). The center of the lesion was eccentric in cases of GCT of the talus and the cuboid (Figure 2). Three cases showed markedly expanded cortex.

Primary surgical procedures included curettage with bone grafting (*n* = 2), curettage followed by ethanol and phenol adjuvant therapy as well as bone grafting (*n* = 3), curettage and bone cementing (*n* = 1), en bloc resection (*n* = 4), and amputation (*n* = 1) (Table I). There were two cases of recurrence, one of which had been treated with curettage and bone grafting as the primary procedure. Although this case was treated with curettage followed by ethanol and phenol adjuvant therapy as a secondary procedure following the first episode of recurrence, the tumor recurred for the third time after 20 months. Metastatic lesions developed in the lungs of one patient, who subsequently died of the disease 13 years after primary surgery.

**Table I.** Some characteristics of 11 patients with small-bone GCT.

Case	Sex	Age	Location	Symptom	Stage	H-E^a^	p63	Initial treatment^b^	Length of follow-up (yrs)	Recurrence	Metastasis
1	F	9	Metacarpal	Swelling with pain	2	B	Weak	Curettage/bone graft (O)	5	No	No
2	M	18	Metacarpal	Pain	3	–	–	En bloc resection (N)	2	Yes	No
3	F	15	Metacarpal	Pain	1	A	Moderate	Curettage/bone cementing	1 1/6	No	No
4	M	30	Metacarpal	Pain	3	C	Weak	En bloc resection	2	No	No
5	M	16	Metacarpal	Pain	2	C	Weak	En bloc resection	1 1/4	No	No
6	F	31	Proximal phalanx (hand)	Swelling	3	–	–	Amputation (N)	13	No	Yes (lung)
7	F	20	Middle phalanx (hand)	Pain	2	A	Strong	Curettage/bone graft	4 1/6	Yes (twice)	No
8	M	21	Talus	Pain	2	B	Weak	Curettage/bone graft (E + P)	4	No	No
9	M	19	Cuboid	Pain	1	A	Moderate	Curettage/bone graft (O)	8	No	No
10	F	60	Metatarsal	Pain	2	A	Moderate	Curettage/bone graft	4 1/3	No	No
11	M	33	Proximal phalanx (foot)	Swelling	2	A	Moderate	En bloc resection	1 1/2	No	No

^a^Characteristics of patients with small-bone GCT as seen by H-E staining: A = The specimen is mostly occupied by compactly gathered mononuclear cells and multinucleated giant cells (typical feature of GCT). B = Secondary ABC change is predominant. C = Detection of the typical feature of GCT is difficult because of the small specimen.

^b^Adjuvant therapy: E + P = ethanol + phenol; N = liquid nitrogen; O = others.

The authors were able to confirm the histological findings of nine cases with the help of a pathologist. During H-E staining, the typical histological characteristics of bone GCT were recognized in five cases, and two showed secondary ABC-like characteristics along with the typical GCT characteristics. Although it was difficult to distinguish the typical characteristics of GCT in two cases, they were not excluded from this study.

**Figure 3.**
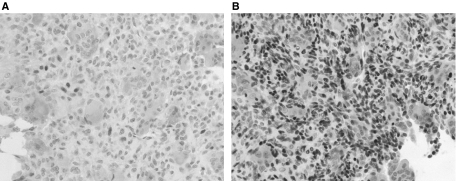
The p63 immunostaining of GCT involving the talus (A) and middle phalanx of the third digit (B). The tumor involving the talus was weakly positive for p63 and did not have any episode of recurrence for 4 years postoperatively, whereas that involving the middle phalanx was highly positive and two episodes of recurrence were noted within 4 years postoperatively.

During p63 immunostaining, five cases were weakly positive (Figure 3A), three were positive, and one was highly positive; the highly positive case had two episodes of recurrence (case 7) (Figure 3B). Five cases of GCT affecting the femur or tibia were subjected to p63 immunostaining for comparison; four were positive and one was highly positive. In addition, GCRG in the metacarpal bone and solid variant of ABC in the humerus demonstrated p63 negativity (data not shown).

## Discussion

Small-bone GCT cases account for only 4% of all GCT cases in Japan (4). Unni (5) and Campanacci (6) reported the incidence of small-bone GCT among all GCTs as 3% in USA and 4.5% in Italy, respectively. These findings indicate that racial differences may not affect the incidence of small-bone GCT.

The main subjective symptom was pain, similar to that observed in conventional GCT cases. A notable radiographic characteristic feature was the marked expansion of the cortical bone, particularly in the short tubular bones (Figure 2). This finding may be explained by occupation of the narrow bone marrow cavity by the tumor in its early stage, resulting in multidirectional expansion of the cortex.

The tendency of small-bone GCT to develop in younger individuals has been reported (7,8). The results of our study are concurrent with these findings, with a 45% incidence (5 of 11 cases) of small-bone GCTs in individuals below 20 years of age; moreover, only 1 patient was above 40 years of age. On the other hand, the incidence of all GCTs in individuals below 20 years of age is 16% in Japan (4).

It has also been reported that GCT of the hand adopts a more aggressive clinical course than that of GCTs occurring in more central locations (9). In contrast, Malawer and Vance reported that GCT in the tarsal bones was not as aggressive and carried a good prognosis (10). In addition, O'Keefe et al. also reported that GCT of the foot was less aggressive (11). Two patients among those who were followed up for over two years in our study had episodes of recurrence; the primary lesion was in the hand in both cases. In addition, one case of primary GCT of the hand developed lung metastasis. The results of our study were therefore concurrent with those of the above-mentioned authors that GCT of the hand has a more aggressive biologic potential.

Dickson et al. and Lee et al. reported that p63 expresses in the mononuclear cells of GCT, and that finding was useful to distinguish GCT from other giant cell-rich tumors such as ABC and chondroblastoma (2,3). All small-bone GCTs except two unexamined specimens in this study showed varying degrees of p63 positivity. On the other hand, GCRG, which is sometimes difficult to distinguish from small-bone GCT (12,13), and the solid variant of ABC in the humerus demonstrated p63 negativity. Therefore, p63 immunostaining appears to be helpful in the differentiation of small-bone GCT from GCRG. Furthermore, two episodes of recurrence after primary surgery were documented for one case of GCT of the hand that was highly positive for p63. Although we were unable to perform p63 immunostaining in the other case of recurrence as well as for the case with lung metastasis, the above-mentioned finding suggests that p63 may be useful not only for the differential diagnosis of GCT but also for prognostication of the aggressiveness of small-bone GCT as a biomarker. Further investigations are needed to confirm the correlation between p63 expression and the clinical behavior of small-bone GCT.

In summary, small-bone GCT tends to develop in individuals below 20 years of age. GCT of the hand may be biologically more aggressive than that occurring in other sites. The p63 immunostaining may be useful for both differential diagnosis and prediction of clinical behavior of small-bone GCTs.
